# Hepatic Tissue Environment in NEMO-Deficient Mice Critically Regulates Positive Selection of Donor Cells after Hepatocyte Transplantation

**DOI:** 10.1371/journal.pone.0100786

**Published:** 2014-06-30

**Authors:** Michaela Kaldenbach, Francisco Javier Cubero, Stephanie Erschfeld, Christian Liedtke, Christian Trautwein, Konrad Streetz

**Affiliations:** Department of Internal Medicine III, University Hospital, RWTH Aachen, Aachen, Germany; Centro de Investigación en Medicina Aplicada (CIMA), Spain

## Abstract

**Background:**

Hepatocyte transplantation (HT) is a promising alternative treatment strategy for end-stage liver diseases compared with orthotopic liver transplantation. A limitation for this approach is the low engraftment of donor cells. The deletion of the I-kappa B kinase-regulatory subunit IKKγ/NEMO in hepatocytes prevents nuclear factor (NF)-kB activation and triggers spontaneous liver apoptosis, chronic hepatitis and the development of liver fibrosis and hepatocellular carcinoma. We hypothesized that NEMO^Δhepa^ mice may therefore serve as an experimental model to study HT.

**Methods:**

Pre-conditioned NEMO^Δhepa^ mice were transplanted with donor-hepatocytes from wildtype (WT) and mice deficient for the pro-apoptotic mediator Caspase-8 (Casp8^Δhepa^).

**Results:**

Transplantation of isolated WT-hepatocytes into pre-conditioned NEMO^Δhepa^ mice resulted in a 6-7 fold increase of donor cells 12 weeks after HT, while WT-recipients showed no liver repopulation. The use of apoptosis-resistant Casp8^Δhepa^-derived donor cells further enhanced the selection 3-fold after 12-weeks and up to 10-fold increase after 52 weeks compared with WT donors. While analysis of NEMO^Δhepa^ mice revealed strong liver injury, HT-recipient NEMO^Δhepa^ mice showed improved liver morphology and decrease in serum transaminases. Concomitant with these findings, the histological examination elicited an improved liver tissue architecture associated with significantly lower levels of apoptosis, decreased proliferation and a lesser amount of liver fibrogenesis. Altogether, our data clearly support the therapeutic benefit of the HT procedure into NEMO^Δhepa^ mice.

**Conclusion:**

This study demonstrates the feasibility of the NEMO^Δhepa^ mouse as an *in vivo* tool to study liver repopulation after HT. The improvement of the characteristic phenotype of chronic liver injury in NEMO^Δhepa^ mice after HT suggests the therapeutic potential of HT in liver diseases with a chronic inflammatory phenotype and opens a new door for the applicability of this technique to combat liver disease in the human clinic.

## Introduction

Orthotopic liver transplantation is currently the only possible cure for patients with acute and end-stage liver diseases. However, due to the complexity and associated morbidity and mortality, the scarcity of donor organs and the need for immunosuppression, other alternatives are being currently considered. The transplantation of isolated hepatocytes has arisen as a potential promising alternative. Here multiple recipients can benefit from one donor liver, in contrast to whole liver transplant.

An extensive deal of work with animal models has demonstrated the repopulation of injured livers after hepatocyte transplantation (HT) by percutaneous or transjugular infusion into the portal vein, or injecting into the splenic pulp or the peritoneal cavity, which is a less invasive procedure compared with liver transplantation [1], [2]. Clinical trials of HT have been initiated at several institutions for acute or chronic liver failure and inherited metabolic disorders [3]. Thereby hepatocyte transplantation has been successfully explored as a vehicle for *ex vivo* gene therapy in children with metabolic disorders [4]. However, a major problem in most HT studies to date has been the limited growth and engraftment of transplanted cells in the recipient organ.

Cirrhosis and hepatocellular carcinoma (HCC) are among the major causes for whole liver transplantation. We have recently described that hepatocyte-specific IKKγ/NEMO knockout (NEMO^Δhepa^) mice represent an excellent liver disease model as it reflects human liver pathogenesis with progression from chronic hepatitis to non-alcoholic steatohepatitis (NASH), liver fibrosis and finally HCC [5], [6]. NEMO is the regulatory subunit of the IKK-complex, which consists of two more subunits – the catalytic forms IKKα and IKKβ [7], [8]. At the canonical pathway, the IKK-complex is activated - through proinflammatory cytokines as TNF-α, viral infection or LPS stimulation – and then, in turn phosphorylates the inhibitor of NF-kB. Once activated NF-kB translocates into the cell nucleus and mediates the expression of target genes associated with cell death, survival, proliferation and inflammation [9], [10].

Hepatocyte specific IKKγ/NEMO-knockout mice develop a phenotype of spontaneous hepatocyte apoptosis, liver fibrogenesis and hepatic neoplasia. Accumulation of extracellular matrix in the liver - which represents the onset of NASH is already present at 12 weeks of age. One year-old livers of NEMO^Δhepa^ mice predominantly consist of fibrotic tissue, steatotic areas and tumorigenic nodules become present [6], [11], [12]. Because of the spontaneous development of chronic liver injury, NEMO^Δhepa^ mice have been regarded as an ideal model for the study of the molecular mechanisms governing hepatocyte transplantation. Thus, we aimed to apply our established model of hepatocyte transplantation [13] in NEMO^Δhepa^ mice. In these animals, activation of TNFα-mediated apoptosis in hepatocytes is a known major key player for the development of chronic liver failure. We thus additionally used donor hepatocytes with a defect in extrinsic apoptosis that were derived from hepatocyte-specific Caspase-8 knockout mice (Casp8^Δhepa^) [11], [14].

Here we show that lack of IKKγ/NEMO creates a hepatic tissue environment that favours efficient liver repopulation after HT. We provide evidence that donor cell engraftment can be further enhanced through use of apoptosis-resistant cells. Our data uncovers the enormous therapeutic potential of this newly established model for the improvement of the engraftment of donor cells after HT and its future clinical applicability.

## Materials and Methods

### Ethics statement

This study was carried out in strict accordance with the recommendations of the Ethics of the regional authorities for nature, environmental and consumerprotection of North Rhine-Westfalia (LANUV - Landesamt für Natur, Umwelt und Verbraucherschutz NRW) Recklinghausen, Germany, and approved by the LANUV Committee (Permit Number: TV10132G1). All surgery was performed under ketamin-hydrochloride/xylazin-hydrochloride anesthesia, and all efforts were made to minimize suffering.

### Housing and generation of mice

Hepatocyte-specific NEMO knockout (NEMO^Δhepa^) and NEMO wildtype (WT, NEMO^loxP/loxP^) mice under control of a postnatal activated albumin promoter were used as recipient mice in a C57BL/6 background [15]. Donor cells were extracted from hepatocyte-specific Caspase-8 deficient mice (Casp8^Δhepa^) and WT (Casp8^loxP/loxP^) littermates [11]. For the generation of donor mice, Casp8^Δhepa^ and Casp8^loxP/loxP^ mice were crossed with human-α1-antitrypsin (hAAT) transgenic mice (C57/Bl6) (Casp8^Δhepa^/hAAT(+); Casp8^loxP/loxP^/hAAT(+)). The expression of the marker protein hAAT is controlled by a α1-antitrypsin-specific promoter to ensure hepatocyte-specific gene expression [13], [16]. At least four mice per group were treated and analysed in parallel for all experiments – that were repeated at least twice. Mice were housed in 12 h light/dark cycles with water and food freely available in the animal facility of the University Hospital RWTH Aachen, and were treated in accordance with the criteria of the German administrative panel on laboratory animal care.

### Bone marrow transplantation (BMT)

Recipient mice were irradiated at the age of 8–10 weeks with a lethal dose of 12 Gray in a cobalt-60-isotope source. Bone marrow donor mice (WT-littermates from hepatocyte donor mice breedings) were euthanized by isoflurane anaesthesia followed by cervical dislocation. The lower extremities were prepared under sterile conditions and the soft tissue was removed from femurs and tibiae and bones were excised. The ends of femur and tibia were cut and the bone marrow was flushed using a 22-gauge needle and Hank's balanced salt solution supplemented with 2% fetal calf serum. To separate the single cells the suspension was filtered through a 70 µm mesh. Cells were washed by centrifugation and resuspended in Hank's balanced salt solution. Finally, cells were suspended in a dilution of 1×10^6^ unfractionated bone marrow cells per 100 µl, which were injected via the tail vein approximately 4 h after irradiation. Recipient mice were housed with antibiotic-supplemented water for 2 weeks.

### Isolation of hepatocytes and hepatocyte transplantation (HT)

Donor mice (hAAT+) were anaesthetised with ketamin-hydrochloride/xylazin- hydrochloride (100 mg/kg). In addition, 100 IE heparin were injected *i.p*. A laparotomy was performed and the inferior vena cava was cannulated. Then, the portal vein was cut and the liver was perfused with Ca^2+^ and Mg^2+^ free Earle's balanced salt solution (EBSS) until the liver had blanched completely. Afterwards, the buffer was changed to EBSS containing Ca^2+^ (1.8 mM) and Mg^2+^ (0.813 mM) and HEPES (10 mM). In the last step, the liver was perfused with EBSS containing Ca^2+^, Mg^2+^ and HEPES and additionally collagenase and an inhibitor of trypsin. The liver was excised and the cells were dispersed in William's Eagle medium. The suspension was filtered through a 70 µm sieve and centrifuged at 500 rpm for 5 min. The cells were washed two times in cold William's Eagle medium, checked for viability via trypan blue staining and finally resuspended to a concentration of 1×10^6^ cells per 100 µl.

Recipient mice were anaesthetised with ketamin-hydrochloride/xylazin hydrochloride (25 mg/kg) and isoflurane inhalation. A lateral abdominal incision was made, the spleen was localised, exposed, and 1×10^6^ cells in a total volume of 100 µl media were injected intrasplenically. Sutured spleens were returned carefully and the skin was closed. Mice were treated with buprenorphinhydrochloride (0.1 mg/kg) to ensure analgesia.

### Blood collection

For retro-orbital bleeding mice were shortly anaesthetised with isoflurane and blood was collected via a glass capillary. Samples were centrifuged at 10.000 rpm for 10 min, aliquoted and serum was stored at −20°C until further analysis.

### ELISA

Serum hAAT expression was analysed with a standard sandwich ELISA. Wells were coated with an anti-hAAT antibody (DiaSorin, Stillwater, Minnesota, USA) in a dilution of 1∶1000 for 1 h at 37°C. Blocking was performed with 5% dry milk powder in TBS-Tween 2 for 1 h at room temperature. The serum samples were incubated in 5% dry milk powder in TBS-Tween20 in a dilution of 1∶1×10^2^ to 1∶1×10^6^, with an incubation time of 2 h at room temperature. An antigen-specific indicator antibody (Research Diagnostics, Inc., Flanders, New Jersey, USA) linked to horseradish peroxidase was used to determine the bound antigen. After applying the substrate 3,3′,5,5′-tetramethylbenzidine-dihydrochloride (Sigma-Aldrich, Steinheim, Germany) and termination of the substrate reaction with sulphuric acid, the absorbance was measured in a fluorescent plate reader at a wavelength of 450 nm. The absorbance values were converted to µg/ml by comparison with a standard curve made from human serum.

### Immunofluorescence stainings

Liver cryosections (5 µm) were air-dried and fixed with ice-cold acetone. After rehydration in phosphate-buffered saline (PBS) the samples were treated with 2 N HCl for 30 min and afterwards neutralised with 0.1 M sodium borate (pH 8.0) for 9 min. The samples were washed in PBS. Antibodies were incubated in 0.2% bovine serum albumin (BSA) and 10% goat serum in PBS (anti-hAAT: 1∶200, RDI; anti-BrdU: 1∶40; Becton Dickinson, Heidelberg, Germany; anti-NK1,1: 1∶100; biolegend, Germany) for 1 h at 37°C or overnight at 4°C, respectively. For the detection of collagen tissue sections were fixed in 4% PFA (Roth, Karlsruhe, Germany) and blocked in 2% BSA in PBS acid. Incubation of the first antibody was performed overnight at 4°C or 1 h at room temperature, respectively (anti-collagen type I: 1∶250; Biotrend, Cologne, Germany; anti-Ki-67: 1∶100 in 0.3% Triton X-100, 5% goat serum in PBS; Nova Castra Laboratories, Newcastle upon Tyne, UK). After washing in PBS Alexa Fluor 488 and/or 594-conjugated secondary antibodies (Molecular Probes/Invitrogen, Karlsruhe, Germany) were used for immunofluorescence detection. The sections were analysed using a fluorescence microscope (Zeiss, Jena, Germany).

### Immunohistochemical staining

Livers from mice were harvested and after fixation with 4% PFA, embedded in paraffin for further histological analysis. Paraffin tissue sections were deparaffinized and rehydrated. For antigen retrieval sections were boiled in sodium citrate trisodium salt dehydrate. After 10 minutes of 1,5% of hydrogen peroxide sections were incubated with the first antibody over night (NEMO, Santa Cruz; 1∶800) in PBT at 4°C. Thereafter the second antibody was incubated for 1 hour at room temperature. Afterwards sections were incubated with streptavidin ABC-alkaline phosphatase and colour was developed with peroxidase DAB followed by hematoxylin counterstaining.

### TUNEL assay

Cryosections (5 µm) were air-dried and fixed with 4% paraformaldehyde at room temperature. After washing with PBS the slides were incubated for 10 min in 3% H_2_O_2_ methanol followed by 2 min incubation in sodium citrate (0.1%). After washing in PBS the substrate mixture was applied according to the manufacturer's instructions (Roche, Mannheim, Germany).

### Gene expression analysis by real-time PCR

Total RNA was extracted from cryopreserved liver tissue using peqGold RNAPure (PeqLab, Erlangen, Germany); 500 ng total RNA was transcribed into complementary DNA with the RT Omniscript kit (Qiagen, Hilden, Germany). Quantification of cDNA expression for specific genes was performed by SybrGreener quantitative PCR Supermix (Invitrogen, Karlsruhe, Germany). Primers are available upon request.

### Quantification and statistics

All numerical results are expressed as mean±SEM and represent data from at least four animals per time point. Calculations via manual counting of positive cells were done with five to 10 high power field/liver. All significant p values were measured by the Student's t test. A value of p<0.05 was considered significant (*p<0.05, **p<0.01, ***p<0.001). Sirius red stainings were photographed using polarized light.

## Results

### Successful selection of donor hepatocytes in NEMO^Δhepa^ recipient mice

The development of liver fibrosis, tumour initiation and progression are among the clinico-pathological features of end-stage chronic liver disease. To test the clinical applicability of liver repopulation in a clinically relevant setting, we chose NEMO^Δhepa^ mice, as an experimental model of chronic liver injury, as recipients for HT.

**Figure 1:**
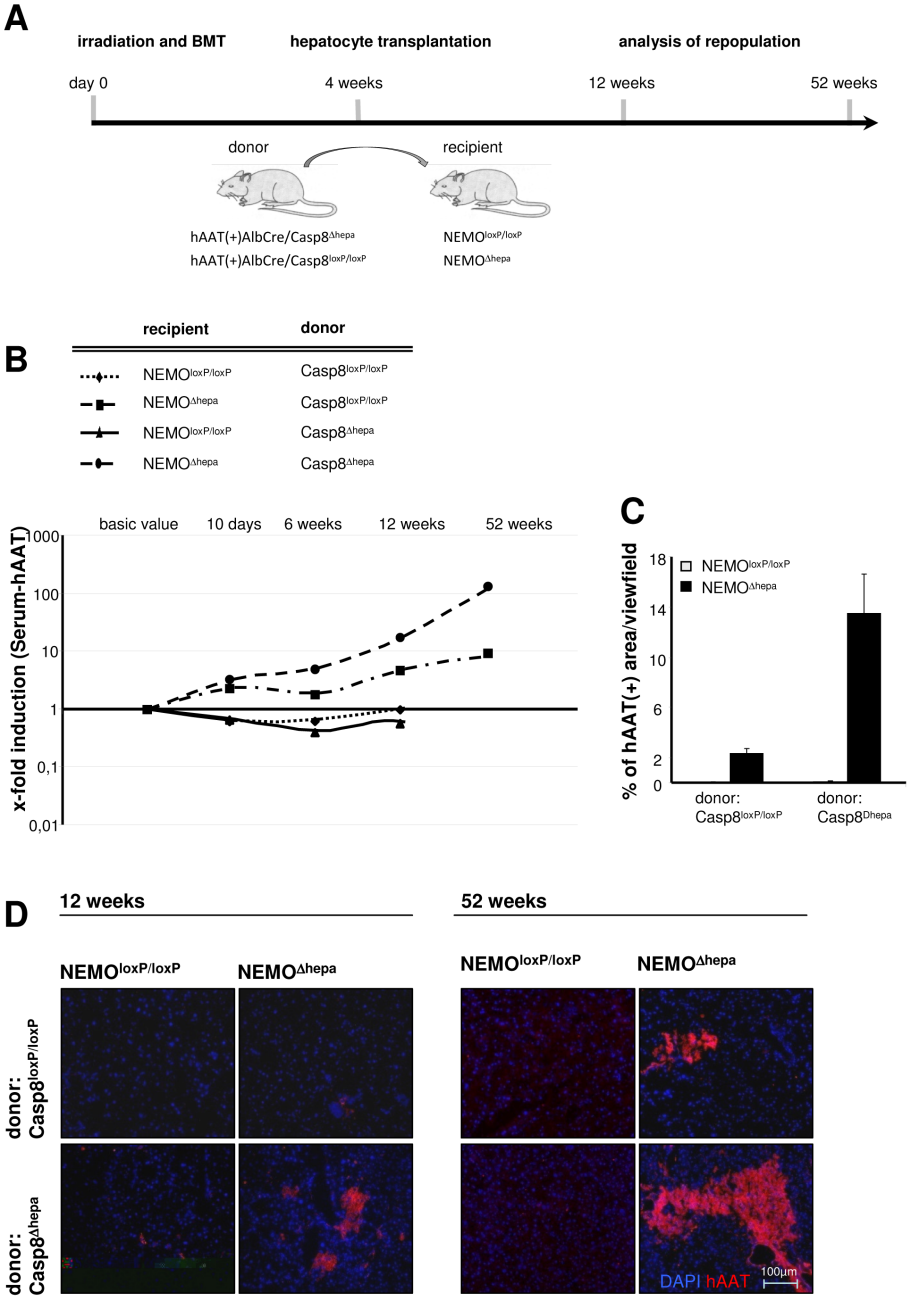
NEMO deficiency in recipient mice favours donor cell selection. (**A**) Hepatocyte transplantation was applied to NEMO^Δhepa^ and WT recipient mice with preceding BMT. Donor cells were derived from WT (Casp8^loxP/loxP^/hAAT(+)) or Caspase-8-deficient (Casp8^Δhepa^/hAAT(+)) mice, respectively. (**B**) Serum hAAT levels were analysed via quantitative ELISA and displayed on a logarithmic scale. Basic values represent serum hAAT level 1d post transplantation and are used as a reference value for the calculation of the relative increase in the amount of donor derived hepatocytes over time. (**C**) Quantitative evaluation of hAAT (+) liver tissue areas 52 weeks after HT. (**D**) Visualization of engrafting donor cells using hAAT immunofluorescence staining. Clusters of donor derived hepatocytes are displayed 12 and 52 weeks after HT (blue: DAPI; red: hAAT(+) cells).

HT was performed in pre-conditioned NEMO^Δhepa^ mice, as previously described [13] (**Fig. 1A**). Recipient mice were exposed to lethal irradiation followed by subsequent bone marrow transplantation (BMT). Altogether four weeks after BMT the transplantation of isolated donor hepatocytes (HT) was performed. Blood samples were taken at day 2, day 10, 6, 12 and 52 weeks after HT. The success of hepatocyte repopulation was determined by measuring serum-hAAT levels in recipient mice by ELISA (**Fig. 1B**) and confirmed by immunostaining (**Fig. 1C** **+D**).

12 weeks after HT we observed a 6-7 fold increase in the number of WT-donor cells after transplantation into pre-conditioned NEMO^Δhepa^ recipient mice, while in WT-recipients no selection was observed. This repopulation continued over a period of 52-weeks (**Fig. 1B**). WT recipients displayed no relevant selection (**Fig. 1C** **+D**). NEMO^Δhepa^ mice are known to display a strong intrinsic activation of TNFα amongst other inflammatory cytokines, which results in enhanced hepatocyte apoptosis [6], [15]. We additionally reasoned that a specific inhibition of the extrinsic apoptosis-inducing pathway in donor cells by using Casp8^Δhepa^ would be beneficial for donor cell selection. Our results clearly show a further enhancement of donor cell selection if Casp8^Δhepa^ hepatocytes were transplanted into NEMO^Δhepa^ recipients (**Fig. 1C** **+D**). Serum hAAT-level of NEMO^Δhepa^ recipients transplanted with donor Casp8^Δhepa^ cells unravelled a 100-fold increase in the amount of donor cells 52 weeks after transplantation (**Fig. 1B**). In contrast, Caspase-8 inhibition in donor cells did not benefit cell selection in WT recipients (**Fig. 1D**). Interestingly, Casp8^Δhepa^ donor cells traceable by hAAT positivity were found clustering in distinct areas of the recipient livers (**Fig. 1D**). The quantification of hAAT-positive tissue in NEMO^Δhepa^ recipient mice showed a partial liver repopulation up to 13% of the parenchyma (**Fig. 1C–D**).

### Hepatocyte transplantation reduces liver injury in NEMO^Δhepa^ mice

Since HT into NEMO^Δhepa^ recipient mice led to a positive selection of donor Casp8^Δhepa^ hepatocytes we questioned whether liver architecture and parenchymal function were altered in the repopulated liver. For this purpose, we performed macroscopical, microscopical and liver function analysis of the NEMO^Δhepa^ recipient livers.

**Figure 2:**
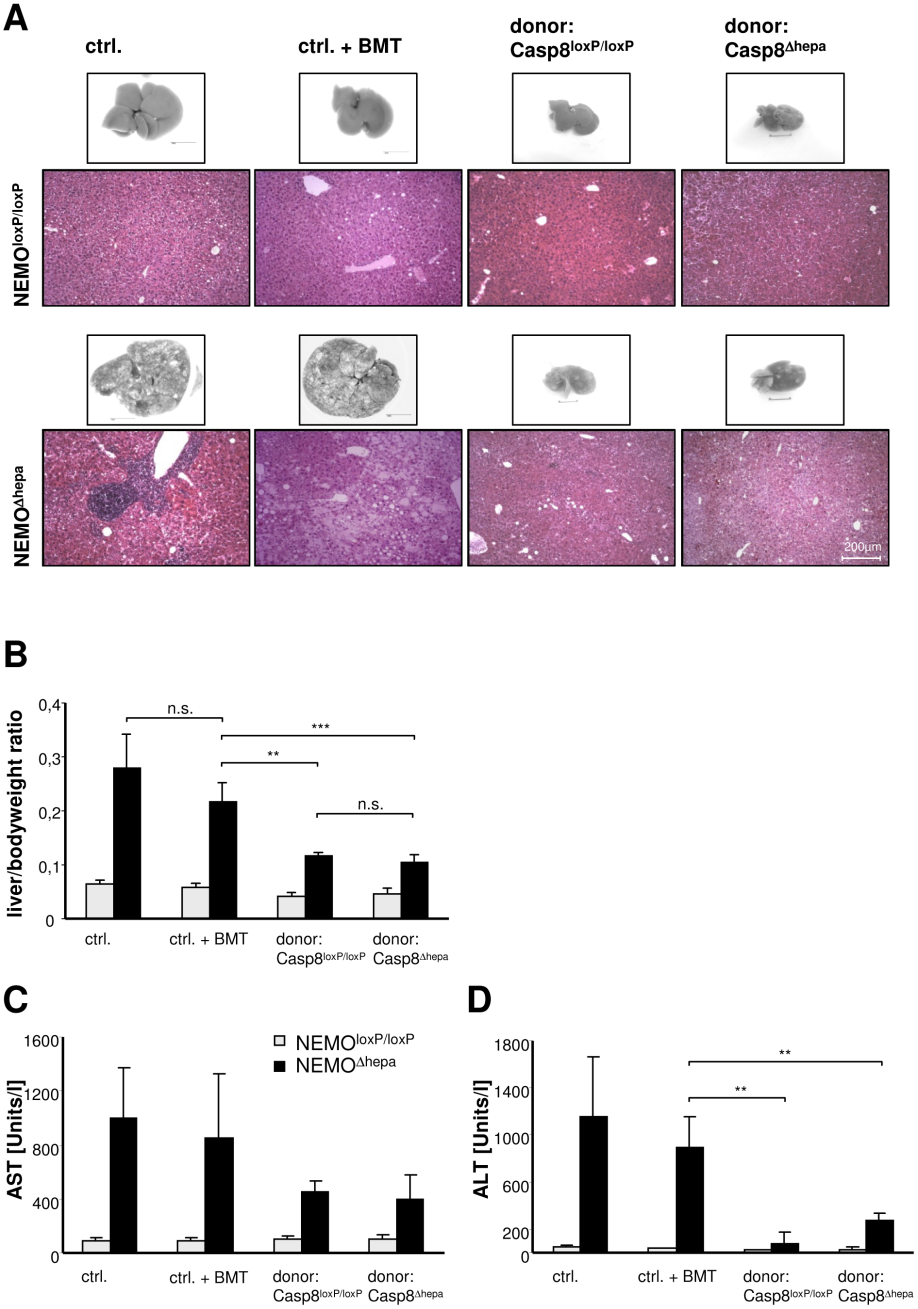
Hepatocyte transplantation in NEMO^Δhepa^ mice results in improved liver function. (**A**) Macroscopical and histological analysis of livers from NEMO^loxP/loxP^ and NEMO^Δhepa^ mice that underwent HT compared to untreated and solely bone marrow transplanted livers. Livers were analysed 52 weeks after HT with either Casp8^loxP/loxP^ (WT) or Casp8^Δhepa^ donor mice in comparison to either naïve or solely BM-transplanted control mice of the appropriate age respectively. Control NEMO^Δhepa^ mice display a progressive fatty degeneration and fibrosis development of their livers over time. This is illustrated by mononuclear cell infiltration, tissue necrosis and hepatocyte ballooning (left side, lower panel). Hepatocyte transplanted NEMO^Δhepa^ mice displayed in this direct comparison less intrahepatic fat accumulation and tissue destruction, thus indicating a beneficial effect of HT in recipient NEMO^Δhepa^ mice. (**B**) Liver *versus* bodyweight ratio, 52 weeks after HT. (**p<0.01, ***p<0.001). (**C, D**) Serum transaminases were measured as an indicator of liver function, showing a clear enhancement in transplanted compared to control mice. (**p<0.01)

Macroscopic analysis of HT recipient livers elicited differences in the morphological appearance after a 52 weeks post-transplantation period (**Fig. 2A**). Microscopical examination indicated the strong presence of infiltrating immune cells in age-matched NEMO^Δhepa^ mice. However, NEMO^Δhepa^ mice that underwent HT exhibited improved liver histology associated with less inflammatory cells and preserved tissue architecture (**Fig. 2A**). NEMO^Δhepa^ mice receiving BMT but no HT displayed a constant increase in body weight and liver size over time. In contrast, matching 52-weeks livers of mice receiving either WT or Casp8^Δhepa^-donor hepatocytes were smaller in their absolute and relative liver size (**Fig. 2A–B**). However, there was no significant difference between mice transplanted with Casp-8^Δhepa^/hAAT(+) or Casp-8^loxP/loxP^/hAAT(+) donor cells, respectively (**Fig. 2A–B**). Moreover, hepatocyte transplanted-NEMO^Δhepa^ recipient animals displayed less transaminases compared to non-hepatocyte transplanted mice of the same age (**Fig. 2C–D**). In summary, HT substantially improved the degree of chronic liver injury in NEMO^Δhepa^ mice.

### Liver fibrogenesis in NEMO^Δhepa^ recipients is reduced after hepatocyte transplantation

**Figure 3:**
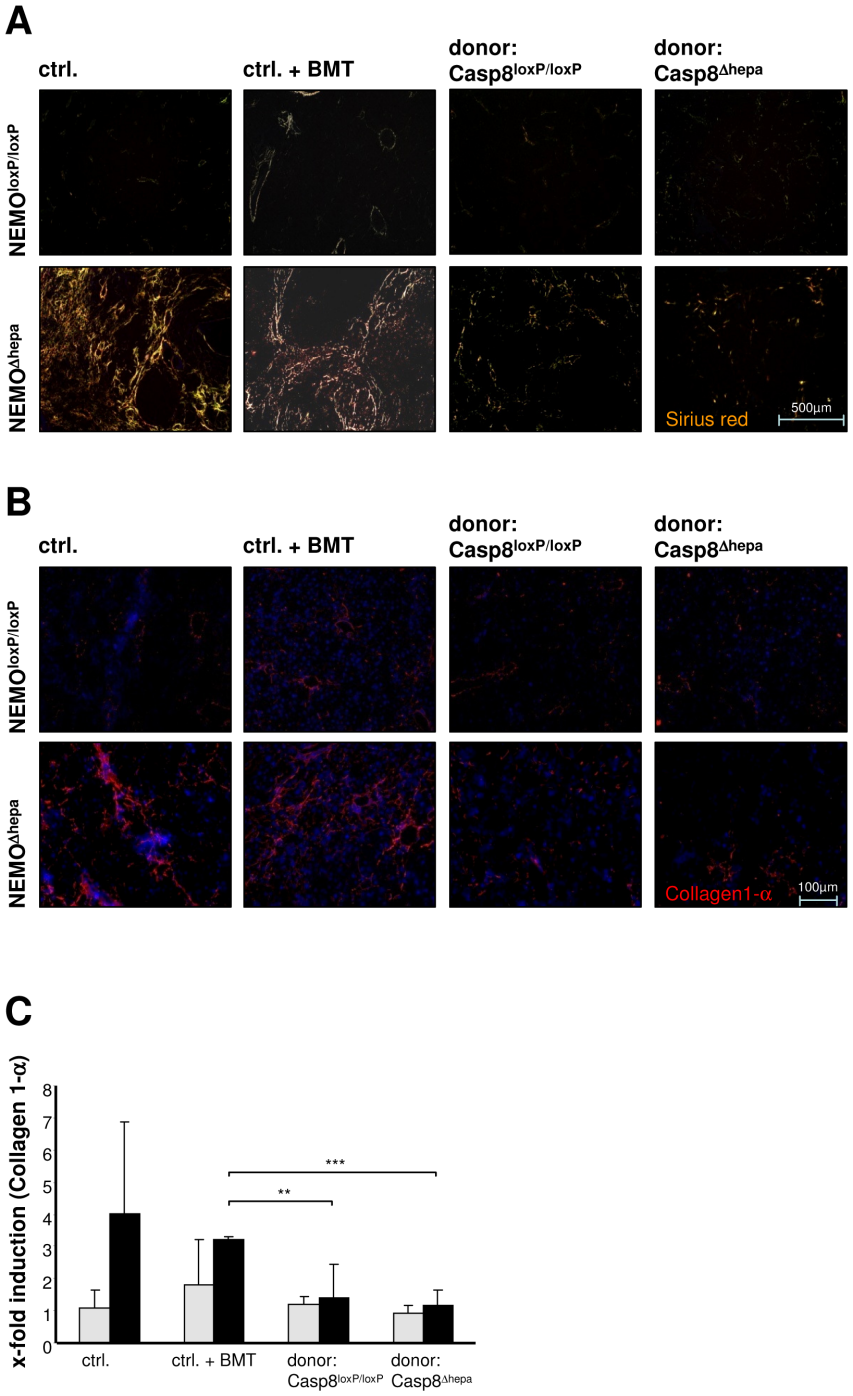
Reduced fibrogenesis in hepatocyte transplanted NEMO^Δhepa^ mice. (**A**) Analysis of Collagen accumulation in transplanted mice via Sirius red staining under polarized light. NEMO^Δhepa^ mice that underwent transplantation with either Casp8^loxP/loxP^/hAAT(+) or Casp8^Δhepa^/hAAT(+) donor cells 52 weeks after HT were compared to age-matched completely untreated control mice as well as to bone marrow-transplanted mice 56 weeks (age matched) after BMT. (**B**) Collagen-1α staining for visualisation of a specific fibrotic collagen subtype (blue: DAPI: red/yellow: Collagen1-α). (**C**) Assessment of Collagen-1α mRNA expression via quantitative realtime PCR. (**p<0.01, ***p<0.001)

Since our previous findings indicated that HT exerts a beneficial effect on NEMO-dependent liver pathogenesis, we next investigated the development of liver fibrosis, which is typically detectable in NEMO^Δhepa^ mice already at the age of 8–13 weeks. Sirius red staining of collagen deposition and immunohistochemical analysis of Collagen-1α fibres displayed a strong signal in age-matched naïve and BMT NEMO^Δhepa^ mice. In contrast, mice which underwent HT showed significantly less collagen accumulation (**Fig. 3A–B**). Quantitative analysis of Collagen-1α mRNA expression by real time PCR displayed a significant reduction in mice subjected to HT independent on the genotype of donor mice (**Fig. 3C**). The onset of liver fibrosis was further graded according to an adapted METAVIR score [17], which revealed an improvement of liver histology in mice subjected to HT as compared to control mice (**Table 1**). Altogether these results suggest that HT attenuates the progression of fibrosis in NEMO^Δhepa^ mice.

**Table 1:** Quantitative histological assessment of mouse livers.

	NEMO^loxP/loxP^	NEMO^Δhepa^
ctrl	F0	F2
ctrl. + BMT	F1	F2-3
donor: Casp8^loxP/loxP^	F0	F1
donor: Casp8^Δhepa^	F0	F1-2

Liver histologies were graded for fibrosis levels according to an adapted METAVIR fibrosis score. F0 =  no fibrosis; F1 =  portal fibrosis without septa; F2 =  portal fibrosis with septa; F3 =  numerous septa without cirrhosis; F4 =  cirrhosis.

### Cell death and hepatocyte proliferation are reduced after HT in NEMO^Δhepa^ mice

**Figure 4:**
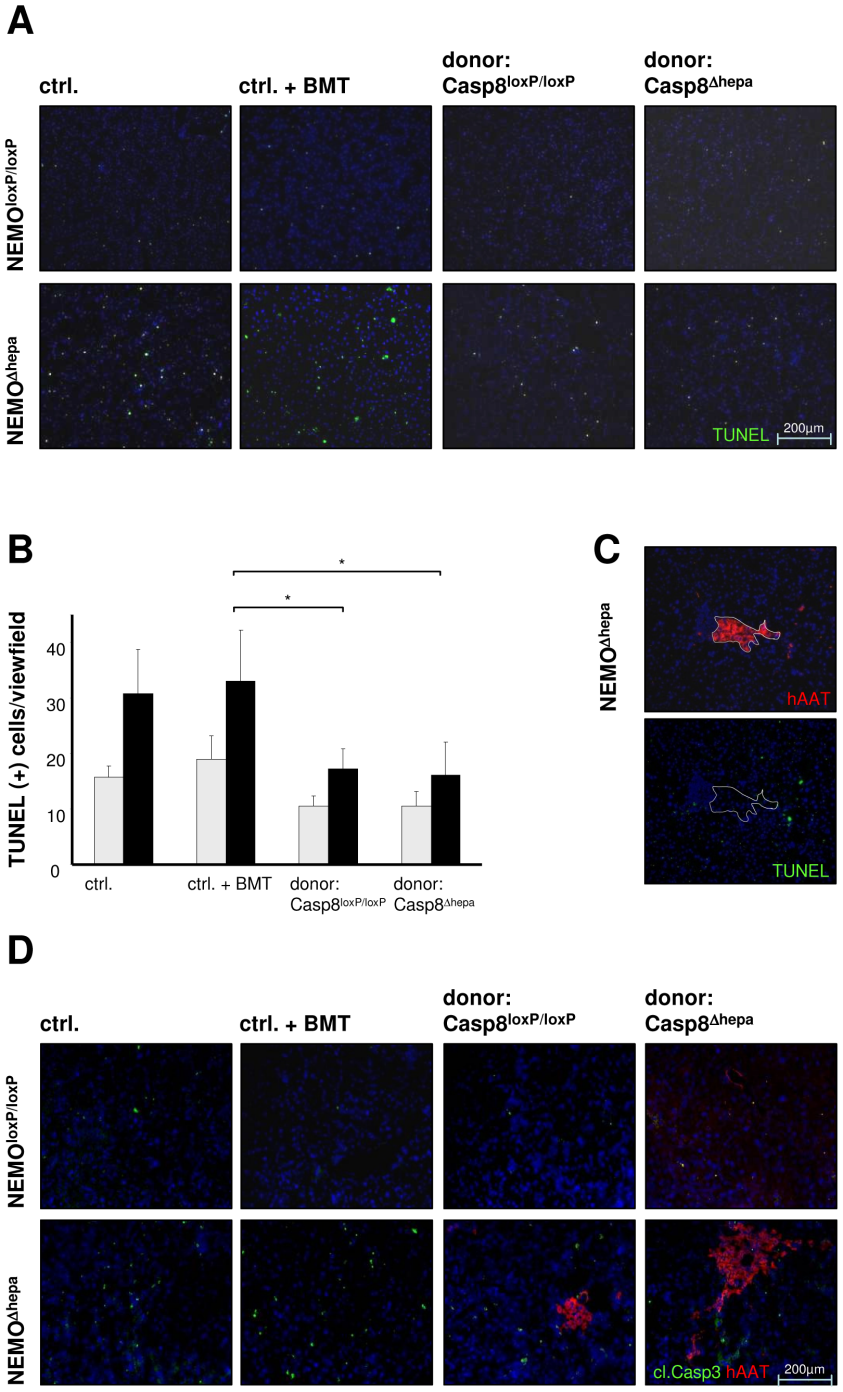
Analysis of hepatic apoptosis. (**A**) TUNEL staining was performed to mark apoptotic cells (blue: DAPI; green: TUNEL (+)). (**B**) Quantification of TUNEL(+) cells in recipients transplanted with Casp8^Δhepa^/hAAT(+) cells or Casp8^Δhepa^/hAAT(+) cells, respectively, compared to naïve control mice and mice subjected to BMT. The total number of apoptotic cells per view field was counted in a 100x magnification. (*p<0.01). (**C**) An analysis of the localization of TUNEL (+) cells was performed via serial sections and immunofluorescence staining of hAAT(+) areas and TUNEL-staining. (**D**) Displayed is a co-immunostaining of cleaved Caspase-3 and hAAT(+) donor cells, which shows an equal distribution of apoptotic cells as compared to figure (A). The use of double-staining reveals, that Caspase-3(+) and hAAT(+) cells are distinct and not overlapping.

**Figure 5:**
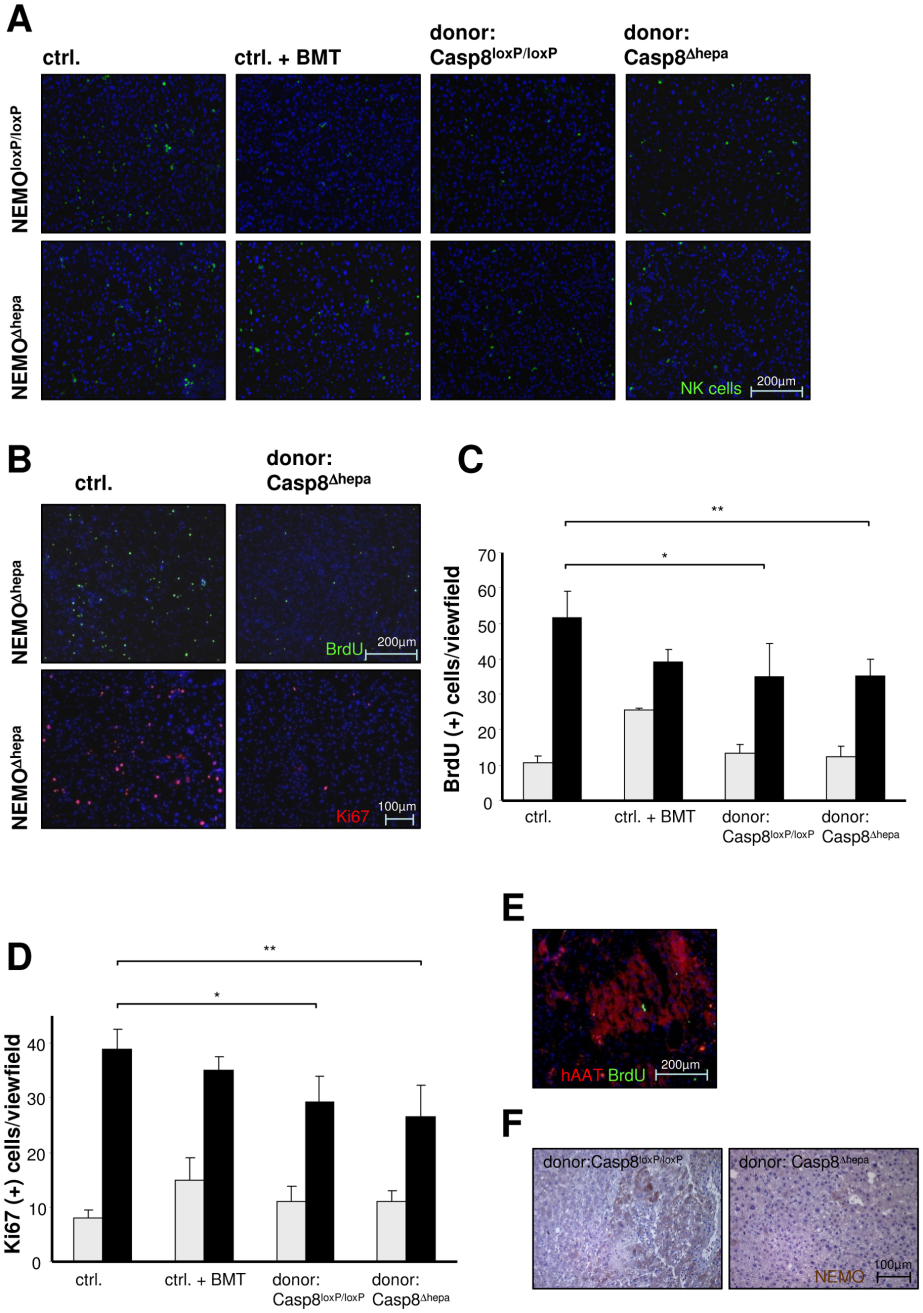
Assessment of NK cell activation and hepatic proliferation. (**A**) NK cell staining was performed in order to address the role of the immune cell response to HT (blue: DAPI; green: NK1.1(+) cells). This shows, that NK-cells are present in a significantly lower number in HT-recipient mice as compared to controls and solely BM-treated animals. (**B**) Visualization of cell proliferation by Ki-67 and BrdU incorporation stainings. (blue: DAPI; green: BrdU(+); red: Ki-67(+)). (**C**) Determination of the total number of BrdU(+) cells by means of BrdU staining in a 100x magnification. (*p<0.05, **p<0.01). (**D**) Quantification of Ki-67 positive signals in immunofluorescent staining using a 200x magnification. (*p<0.05, **p<0.01). (**E**) For localization of proliferating cells, double immunofluorescence staining of BrdU and hAAT was performed (blue: DAPI; red: hAAT(+) cells; green: BrdU(+) cells). (**F**) DAB-staining of NEMO protein in NEMO^Δhepa^ mice transplanted with Casp8^loxP/loxP^/hAAT(+) or Casp8^Δhepa^/hAAT(+) donor cells, respectively (DAB in brown: NEMO protein). This clearly shows the presence of NEMO(+) hepatocytes in the surrounding NEMO-deficient (NEMO^Δhepa^) liver tissue.

Enhanced TNF-α expression leads to massively increased programmed cell death in NEMO^Δhepa^ mice [12], [15], [18]. Therefore, we investigated the onset of apoptosis in HT-recipients using TUNEL-assay. Untreated old (age matched) NEMO^Δhepa^ mice exhibited a large number of apoptotic cells (**Fig. 4A**). NEMO^Δhepa^ mice subjected to BMT without HT showed similar apoptotic activity compared to untreated mice (**Fig. 4A**). In contrast, lower rates of apoptotic cells were found in NEMO^Δhepa^ mice 52 weeks after HT. Quantification of TUNEL(+) cells confirmed a significant decrease of cell death in NEMO^Δhepa^-hepatocyte transplanted mice (**Fig. 4B**). The decrease in apoptosis observed in NEMO-knockout mice however was independent of the donor cell-genotype (**Fig. 4A–B**). In order to determine the distribution of apoptotic cells in recipient mice, TUNEL and anti-hAAT immunofluorescence stainings were performed on serial sections in liver tissue, 12 weeks after HT. This analysis showed that apoptosis was mainly restricted to resident cells instead of donor derived hepatocytes (**Fig. 4C**). Cleavage of the effector Caspase-3 plays a relevant role in the induction of apoptosis [11]. Therefore a co-staining of activated Caspase-3 and hAAT(+) donor cells was performed (Fig. 4D). This experimental approach confirmed the aforementioned results, since a reduced cleaved caspase-3 became evident in mice that underwent HT compared with untreated control mice and mice exclusively subjected to BMT. The further examination of the co-staining clearly showed that there is no overlap of activated caspase-3(+) and hAAT(+) donor cells (**Fig. 4D**). This might indicate that donor-derived hepatocytes are not undergoing apoptosis. Apoptosis induction in NEMO^Δhepa^ mice has been shown to correlate with NK-cell activation [15]. Consistent with the reduction of apoptosis observed by TUNEL and cleaved Caspase-3 stainings, the activation of NK cells after HT was diminished as well (**Fig. 5A**).

Cell death is often followed by compensatory proliferation of hepatocytes. Thus, we next investigated whether the effect of HT on NEMO^Δhepa^ mice was restricted to fibrosis and apoptosis, or if other parameters such as hepatocyte proliferation, possibly triggering HCC-development were affected. The analysis of cell proliferation using BrdU incorporation and Ki-67 immunostaining clearly showed decreased numbers of proliferating liver cells in mice, 52 weeks after HT in NEMO^Δhepa^ mice compared to untreated controls (**Fig. 5B**). Quantification of BrdU- and Ki-67-positive cells confirmed the decrease in overall cell proliferation in NEMO^Δhepa^ livers after HT (**Fig. 5C–D**). However, double immunofluorescence staining revealed that BrdU-positive cells were indeed hAAT(+) cells, indicating that proliferation in mice that underwent HT was predominantly evident in cells descendant from transplanted donor cells (**Fig. 5E**). This finding was further confirmed by the visualization of the NEMO protein on paraffin sections in NEMO^Δhepa^ mice. Our results showed clusters of NEMO positive areas, indicative of the engraftment of donor cells (**Fig. 5F**).

## Discussion

Orthotopic liver transplantation in the clinic is limited due to the increasing shortage of donors and the ample margin of pathologies - from end-stage liver fibrosis or the occurrence of HCC - where the procedure is indicated. Hepatocyte transplantation is currently considered as a promising alternative, especially once the yield and outcome of this technique can be improved. In our previous studies we demonstrated the feasibility of HT and identified responsible factors for donor cell selection in this model [13], [19].

First, we observed a significant selection advantage for WT-hepatocytes after transplantation into NEMO^Δhepa^ livers, very likely to be attributed to the extensive structural changes observed in livers of these animals over time. Besides the progressive hepatic fibrosis, NEMO^Δhepa^ livers also comprise a strong and persistent inflammation, which is among others – characterised by constantly activated TNF-α. Thus the intrinsic regenerative approach in livers of NEMO^Δhepa^ mice is accompanied by a permanent induction of apoptosis in parenchymal liver cells. This induction of apoptosis is also present in the transplanted WT-hepatocytes and counterbalances the compensatory proliferation. Indeed, this mechanism might explain the reason for which transplanted cells are not selected at a higher rate as one might expect. To overcome this potential problem, we used apoptosis-resistant Casp8^Δhepa^ donor mice. Casp8^Δhepa^ hepatocytes are characterised by their resistance against receptor mediated death signalling which leads to apoptosis [11]. Moreover, Casp8^Δhepa^-mice were recently shown to have an earlier onset of DNA-synthesis during liver regeneration. This is among others mediated through a premature activation of the transcription factor NF-κB [20] and likely additionally contributes to the observed advantageous – up to ten-fold higher selection rate - of Casp8^Δhepa^-donor hepatocytes in NEMO^Δhepa^ mice. Moreover, the beneficial effect Casp8^Δhepa^ donor cells lasted throughout the entire time- frame of the experiments.

Unexpectedly, we did not achieve liver repopulation in unconditioned NEMO^Δhepa^ livers, independent of the time when HT was performed, either in young (12 weeks) or aged-mice (52 weeks, data not shown). In our previous reports, we showed that BMT usually is required for successful repopulation after HT [19], [21]. These data were confirmed in NEMO^Δhepa^ mice, where we observed selection of transplanted hepatocytes only after preceding-BMT. This suggests that the phenotype of NEMO^Δhepa^ mice might not only depend on a gene-defect in hepatocytes, but it is also likely that immune cells play an essential role in this process. Based on these findings lethal irradiation and subsequent BMT were performed 4 weeks before HT, which resulted in a constant selection of the transplanted hAAT(+) cells (Figure 1A, B). NEMO^loxP/loxP^ recipient mice (WT) however, did not display a significant repopulation of the transplanted cells, even though being subjected to BMT. Thus, the onset of fibrosis development in NEMO^Δhepa^ recipient mice most likely provides a better niche for the proliferation of transplanted hepatocytes. Inflammation, triggering intrinsic regeneration and thus cell proliferation in NEMO^Δhepa^ mice might certainly be an additional factor for the observed cell selection of transplanted cells. Due to the complexity of distinguishing between these phenomena we can only assume that the constant selection of donor cells is due to progressive fibrogenesis and associated inflammation. Under both conditions transplanted healthy donor cells seem to have a proliferative advantage.

Transplantation of bone marrow derived-cells has been shown to reduce CCl_4_ –induced liver injury in mice [22]. However, the impact of irradiation and BMT for liver fibrosis is still a matter of controversy. Different studies showed hardly any improving effect of BMT to fibrosis development [23]. To solve this problem, we included a second control group that underwent BMT and was sacrificed at 52 weeks of age. Our results showed a similar phenotype to naive control mice, meaning that, in our experimental setting, BMT has no correcting effect in liver fibrosis development concomitant with a recent report [23].

Both syn- and allogeneic transplantation of isolated hepatocytes have been shown to actually correct liver functions in yet irreversibly cirrhotic livers and prolong survival in mice, despite the loss of cell function over time [24], [25]. Here, our experimental model demonstrates successful graft function and ongoing selection of donor cells up to 52 weeks after transplantation.

In parallel, we observed that NEMO^Δhepa^ mice show an improved phenotype over time if they underwent HT. In our experiments NEMO^Δhepa^ mice exhibited significantly lower transaminase levels and less severe histopathological changes 52 weeks after HT compared with age-matched non-transplanted mice or mice subjected to BMT only. This was observed in both -animals transplanted with Casp8^loxP/loxP^/hAAT(+) or Casp8^Δhepa^/hAAT(+) cells. We believe that the reduced inflammatory response is closely linked to a general effect related to the engraftment and proliferation of adult donor hepatocytes. Most likely hepatocyte transplantation induces beneficial immune-modulatory changes, regardless of the genotype of donor cells [26].

Thus the presence of the transplanted wt-cells with intact NF-κB-activation is beneficial for the progression of liver disease in NEMO^Δhepa^ mice. In fact, it is known that liver fibrosis can be experimentally reversed [27]. Here, we report a delay in the development of liver fibrosis but incomplete regression after HT due to the fact that NEMO-deficient mice suffer progressive structural changes.

Apoptotic activity has been regarded as a possible factor for triggering fibrosis progression [12]. Our results evidence that mice subjected to HT exhibit reduced apoptosis. Interestingly, BMT does not interfere with apoptosis since control mice subjected to BMT show a comparable number of apoptotic cells than naïve control mice.

In summary, within the present study we demonstrate that the NEMO^Δhepa^ mouse is a versatile model to study technical and biological aspects related to HT. Indeed there are several more studies showing effective and in part higher levels of donor cell repopulation. The fumaryl acetoacetat hydrolase mouse (Fah^-/-^) - representing an ideal model of metabolic disorders - was shown to be cured from hereditery tyrosinemia by transplantation of only very few hepatocytes [28]. Chemical preconditiong via retrorsine - a cell cycle inhibiting pyrrolizidin alkaloid thus inducing chronic liver injury - resulted in strong liver mass replacement as well [29]. However, a genetic model triggering liver fibrogenesis was not applied to HT up-to-date. Therefore the use of NEMO^Δhepa^ mice as recipients for HT represents a promising novel experimental model. Donor cell engraftment after HT may here function as a modulator of the spontaneous progression of liver fibrosis. This seems to represent a special feature of the NEMO^Δhepa^ mouse, which cannot be investigated in other HT-models to the same extent. Notwithstanding, the expansion of liver repopulation by transplanted hepatocytes is rather modest (up to 13%) which clearly suggests that other factors (growth factors e.g. HGF, cytokines like IL-10,) might help to ameliorate the progression of chronic liver injury in NEMO^Δhepa^- transplanted mice. This could explain the observed improvement in the NEMO^Δhepa^ phenotype which became also apparent in recipients of WT-donors cells, displaying a less repopulation efficacy. Similar effects are described already in experiments involving transplation of stem cells in hepatic injury models [30], [31]. The transplantation efficacy is a current matter of debate since it varies a lot among different repopulation models and even between individual experiments. Most importantly, in this study we show the spontaneous repopulation of NEMO^Δhepa^ recipient mice with transplanted hepatocytes, whereas in other models often chemical pre-treatments must by applied [29], [32]. Thus the particular value of the NEMO^Δhepa^ model can be the fact that it allows the investigation of modifications in the cell transplant setting that do have only minor – otherwise not clearly observable - effects on cell selection. Liver repopulation in NEMO^Δhepa^ takes place continuously and lasts at least for 52 weeks after HT and even most likely throughout the entire lifespan of the mouse. Hence, our findings bring some hope so that transplantation of hepatocytes might be a potential option for the treatment of patients with fibrotic liver diseases.
